# Expression of basement membrane genes and their prognostic significance in clear cell renal cell carcinoma patients

**DOI:** 10.3389/fonc.2022.1026331

**Published:** 2022-10-24

**Authors:** Junyue Tao, Xiao Li, Chaozhao Liang, Yi Liu, Jun Zhou

**Affiliations:** ^1^ Department of Urology, The First Affiliated Hospital of Anhui Medical University, Anhui Medical University, Hefei, China; ^2^ Institute of Urology, Anhui Medical University, Hefei, China; ^3^ Anhui Province Key Laboratory of Genitourinary Diseases, Anhui Medical University, Hefei, China

**Keywords:** clear cell renal cell carcinoma, basement membrane (BM), gene expression profile, prognostic biomarkers, gene expression analysis

## Abstract

**Background:**

Clear cell renal cell carcinoma (ccRCC) is a malignant tumor with limited treatment options. A recent study confirmed the involvement of basement membrane (BM) genes in the progression of many cancers. Therefore, we studied the role and prognostic significance of BM genes in ccRCC.

**Methods:**

Co-expression analysis of ccRCC-related information deposited in The Cancer Genome Atlas database and a BM geneset from a recent study was conducted. The differentially expressed BM genes were validated using quantitative reverse-transcription polymerase chain reaction (qRT-PCR). Least absolute shrinkage and selection operator regression and univariate Cox regression analyses were performed to identify a BM gene signature with prognostic significance for ccRCC. Multivariate Cox regression, time-dependent receiver operating characteristic, Kaplan–Meier, and nomogram analyses were implemented to appraise the prognostic ability of the signature and the findings were further verified using a Gene Expression Omnibus dataset. Additionally, immune cell infiltration and and pathway enrichment analyses were performed using ImmuCellAI and Gene Set Enrichment Analysis (GSEA), respectively. Finally, the DSIGDB dataset was used to screen small-molecule therapeutic drugs that may be useful in treating ccRCC patients.

**Results:**

We identified 108 BM genes exhibiting different expression levels compared to that in normal kidney tissues, among which 32 genes had prognostic values. The qRT-PCR analyses confirmed that the expression patterns of four of the ten selected genes were the same as the predicted ones. Additionally, we successfully established and validated a ccRCC patient prediction model based on 16 BM genes and observed that the model function is an independent predictor. GSEA revealed that differentially expressed BM genes mainly displayed significant enrichment of tumor and metabolic signaling cascades. The BM gene signature was also associated with immune cell infiltration and checkpoints. Eight small-molecule drugs may have therapeutic effects on ccRCC patients.

**Conclusion:**

This study explored the function of BM genes in ccRCC for the first time. Reliable prognostic biomarkers that affect the survival of ccRCC patients were determined, and a BM gene-based prognostic model was established.

## Introduction

There are over 300,000 new cases of clear cell renal cell carcinoma (ccRCC), acounting for the most prevalent subtype of renal malignancy, reported worldwide in 2020 (1). In recent years, several alternative treatments, such as surgery, immunotherapy, and other targeted therapy, have been applied for ccRCC patients (2). For patients with ccRCC at early localized stage, surgery remains the first-line therapy; yet 30% of them meet post-surgery recurrence (3). Despite encouraging achievements in immunotherapy and targeted therapy, the five-year survival probability for metastatic ccRCC has only improved by 11.7% (4–6). Therefore, exploring the mechanism and mining potential biomarkers of ccRCC have become the focus of kidney cancer research.

The basement membrane (BM) is the oldest extracellular matrix (ECM) in animals, bordering all cells, including the epithelium and endothelium (7). The BM core structural components belong to the laminin family, collagen IV, heparan sulfate proteoglycans, nidogens, and perlecan (8). Utilizing these basic components, the basement membrane plays a vital biological role in the body, resisting mechanical stress, determining tissue morphology, establishing a diffusion barrier, and providing an environment for guiding cell polarity, differentiation, migration, and survival (9–12). Over 20 BM gene mutations form the basis of human diseases, highlighting their diverse and vital functions (13). As targets of autoantibody attack in immune diseases, deficiencies in the expression and turnover of BM proteins are crucial causative factors in cancer, fibrosis, and diabetes (14–16). Collagen type IV, alpha-6 (*COL4A6*) is a BM gene encoding the a6 chain of collagen IV. *COL4A6* is highly downregulated in prostate cancer, and its deletion can promote prostate cancer progression and metastasis by activating the p-focal adhesion kinase (FAK)/matrix metallopeptidase 9 (MMP-9) signaling pathway (17). Nephronectin (NPNT) has also been shown to be a key regulator of tumor metastasis (18). Huang et al. reported that in metastatic hepatocellular carcinoma, overexpressed NPNT could promote malignant progression through transcriptional regulation of the FAK/phosphatidylinositol 3−kinase (PI3K)/protein kinase B (AKT) signaling cascade (19). Peroxidasin (PXDN) is a BM-associated protein with peroxidase activity that promotes the proliferation, invasion, and migration of ovarian cancer cells, and PXDN overexpression has been correlated with an unfavorable prognosis (20). A disintegrin and metalloproteinase with thrombospondin motifs (ADAMTS) protein is a zinc metalloendopeptidase whose substrates are mostly ECM components associated with multiple malignant phenotypes, including cancer progression and metastasis (21–23).

However, we currently lack systematic studies on the relationship between BM genes and ccRCC. Herein, we used bioinformatics analyses to determine the prognostic significance of the BM gene family in ccRCC and the related mechanisms affecting prognosis to provide a reference for treating ccRCC.

## Materials and methods

### Acquisition of data and identification of differential expression BM genes

The gene expression and related clinical characteristics of 539 ccRCC and 72 noncancerous renal tissue specimens were acquired from The Cancer Genome Atlas (TCGA) (https://portal.gdc.cancer.gov). In a recent study of BM genes, we downloaded a set of 224 BM genes (24). We also downloaded GSE46699, GSE22541, and GSE29609 datasets of GEO (https://www.ncbi.nlm.nih.gov/geo/), totaling 128 ccRCC organization information. The downloaded data were normalized with the corresponding R package, and the R package “limma” (25) was utilized for identification of the differentially expressed BM genes (DEGs). DEGs having a |log2 fold change (FC)| > 1 and an adjusted *P <*0.05 were considered for subsequent analysis.

### Verification of the expression levels of DEGs

Quantitative reverse transcription-polymerase chain reaction (qRT-PCR) was performed to test the transcript abundances of the DEGs. TRIzol (Invitrogen, Shanghai, China) reagent was employed for isolation of total RNA from the HEK-293 and 786-O cells. The primers used to test the expression of selected DEGs are listed in **Supplementary Table 1**. The PCR program was 94°C 3 min, 22 rounds of 94°C 30 s, 55°C 30 s, and 72°C 30 s, and 72°C 5 min. All the reactions were conducted in triplicate.

### Construction and validation of the BM gene signature

Genes associated with the prognosis of ccRCC were identified by univariate Cox regression from the DEGs with the R package “glmnet” (26). We also carried out a least absolute shrinkage and selection operator (LASSO)-penalized Cox regression analysis for construction of a prognostic risk model. Each screened BM gene’s risk score was determined as follows:


Risk score = (Coef 1×mRNA1 expression) + (Coef 2×mRNA2 expression) + (Coef n×mRNAn expression)

(27)

Coef represents the coefficient of the LASSO-Cox analysis for a specific mRNA. The median risk score was calculated, based on which patients with ccRCC were classified to a high- or low-risk group. For evaluation of the model’s prediction ability, we conducted a time-based receiver operating characteristic (ROC) analysis of the model with the survival ROC package (28). Three downloaded GEO datasets were used as verification sets.

### Identification of independent prognostic indices and establishment of the predictive nomogram

Correlations between BM gene expression features and clinical variables were also determined. Univariate and multivariate Cox regression analyses combined with other clinical variables were conducted to test the performance of the our prognostic BM gene signature. The nomogram was established through clinical variables and the BM gene-based model risk score to evaluate the 1-year, 3-year and 5-year OS in ccRCC patients. The prediction effect of the nomogram was assessed by measuring the concordance index and plotting a calibration curve.

### Functional annotation and gene set enrichment analysis

Kyoto Encyclopedia of Genes and Genomes (KEGG) pathway enrichment analysis and Gene Ontology (GO) annotation were carried out for high- and low-risk populations by utilizing the R package “ClusterProfiler” (29). *P <* 0.05 was deemed to signify statistical significance.

Through GSEA, we explored the potential mechanisms underlying low-risk and high-risk populations from a molecular biology perspective. *P <* 0.05 and FDR < 25% were considered significantly enriched.

### Analysis of the infiltration levels of immune cells

Based on the features of B cell-specific long non-coding RNAs, we used the MCP-counter, CIBERSORT-ABS, EPIC, XCELL, TIMER, and QUANTISEQ algorithms to evaluate the differences in immune cell infiltration levels between low-risk and high-risk populations. The expression of some immune checkpoints in the two groups was examined to explore possible immune checkpoint blocking therapies, such as *LAG3*, *ICOS*, *TIGIT*, *CTLA4*, *PDCD1*, and *BTLA*. Additionally, the association between 16 BM genes and immune cells was determined using the TIMER database (http://cistrome.shinyapps.io/timer/), which deepened our knowledge of the effects of BM genes on ccRCC.

### Identification of potential small molecule drugs

Molecular identification of drugs is a crucial link in drug detection. The Drug Signatures Database (DSigDB) was searched for candidate drugs implicated with the differential expression of the BM genes. The Enrichr platform (https://amp.pharm.mssm.edu/Enrichr/) served as the access path for the DSigDB database.

### Statistics analysis

R software (version 4.0.5) was utilized for analysis of statistical data. Wilcoxon test was utilized to examine differences between groups, and *P <*0.05 was deemed to indicate statistical significance.

## Results

### Establishment and validation of the BM gene‑based model

**Figure 1:**
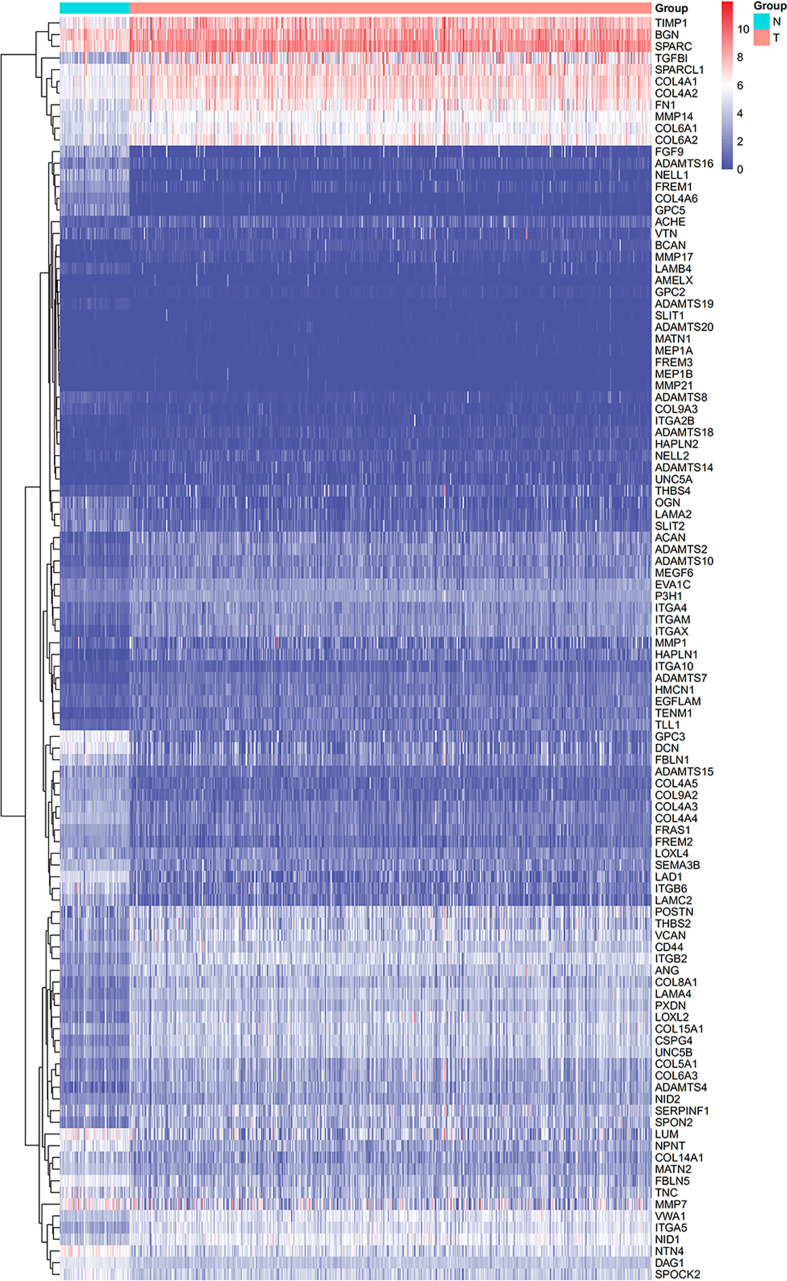
The heatmap displaying the DEGs.

**Figure 2:**
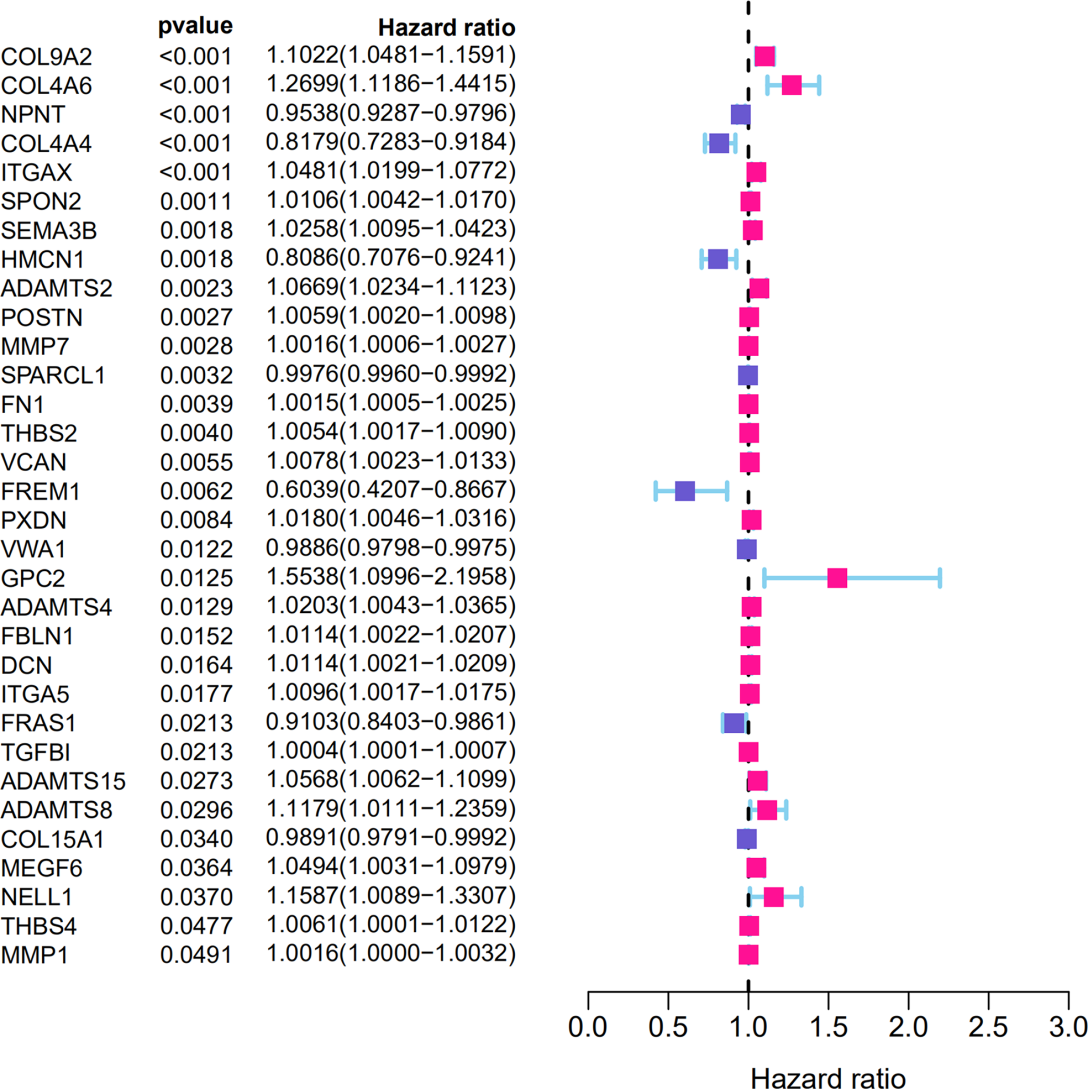
The BM genes with prognostic significance in ccRCC.

**Figure 3:**
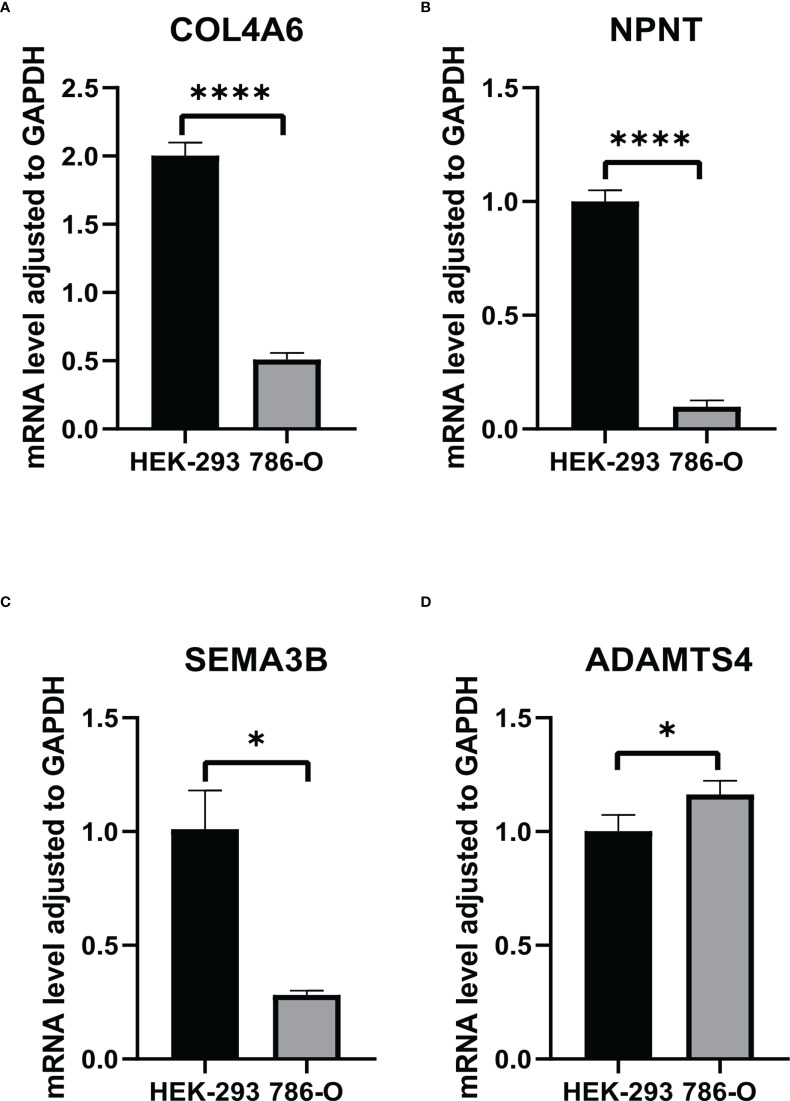
The RNA levels of **(A)** COL4A6, **(B)** NPNT, **(C)** SEMA3B, **(D)** ADAMTS4 in HEK-293 and 786-O cells. "*" represents P < 0.05, "****" represents P < 0.0001.

From the TCGA-KIRC dataset, 108 BM genes were identified to be differentially expressed compared to that in normal kidney tissues. These DEGs included 39 downregulated and 69 upregulated BM genes (**Figure 1**). Subsequently, we implemented univariate Cox regression analysis for identification of the differentially expressed genes with prognostic significance. The results revealed that 32 genes had prognostic values (**Figure 2**), and the qRT-PCR analyses demonstrated that four of the ten genes tested were expressed as predicted (**Figure 3**).

Subsequently, the top 20 genes were selected according to their significance, and a LASSO-Cox regression analysis was carried out. A risk model involving 16 genes (*COL9A2*, *COL4A6*, *NPNT*, *COL4A4*, *ITGAX*, *SEMA3B*, *HMCN1*, *ADAMTS2*, *MMP7*, *FN1*, *VCAN*, *FREM1*, *PXDN*, *VWA1*, *GPC2*, and *ADAMTS4*) was successfully constructed. The risk score was measured with coefficients for the 16 BM genes as follows (**Table 1**): Risk score = (0.0788 × *COL9A2* mRNA level) + (0.1435 × *COL4A6* mRNA level) + (−0.0198 × *NPNT* mRNA level) + (−0.0378 × *COL4A4* mRNA level) + (0.0082 × *ITGAX* mRNA level) + (0.0027 × *SEMA3B* mRNA level) + (−0.1336 × *HMCN1* mRNA level) + (0.0221 × *ADAMTS2* mRNA level) + (0.0003 × *MMP7* mRNA level) + (0.0001 × *FN1* mRNA level) + (0.0020 × *AN* mRNA level) + (−0.0392 × *FREM1* mRNA level) + (0.0103 × *PXDN* mRNA level) + (-0.0075 × *VWA1* mRNA level) + (0.2294 × *GPC2* mRNA level) + (0.0090 × *ADAMTS4* mRNA level).

**Table 1:** The list of signature genes and their coefficients.

Gene symbol	Coefficient
COL9A2	0.0788
COL4A6	0.1435
NPNT	-0.0198
COL4A4	-0.0378
ITGAX	0.0082
SEMA3B	0.0027
HMCN1	-0.1336
ADAMTS2	0.0221
MMP7	0.0003
FN1	0.0001
VCAN	0.0020
FREM1	-0.0392
PXDN	0.0103
VWA1	-0.0075
GPC2	0.2294
ADAMTS4	0.0090

**Figure 4:**
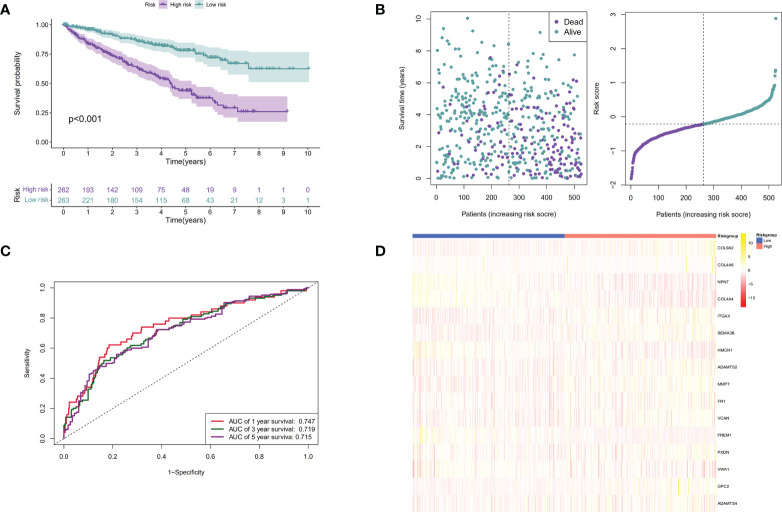
Establishment of the BM genes-based prognostic signature based on the TCGA dataset. **(A).** The Kaplan-Meier (K-M) curves of low-risk and high-risk ccRCC patients in the TCGA dataset; **(B).** The time-dependent ROC curves displaying the 1-year, 3-year, and 5-year OS of ccRCC patients in the TCGA dataset; **(C).** Survival distributions of the TCGA dataset determined according to the median risk score; **(D).** Heatmap displaying the divergences between low- and high-risk patients of 16 signature genes in the prognostic model for the TCGA dataset.

**Figure 5:**
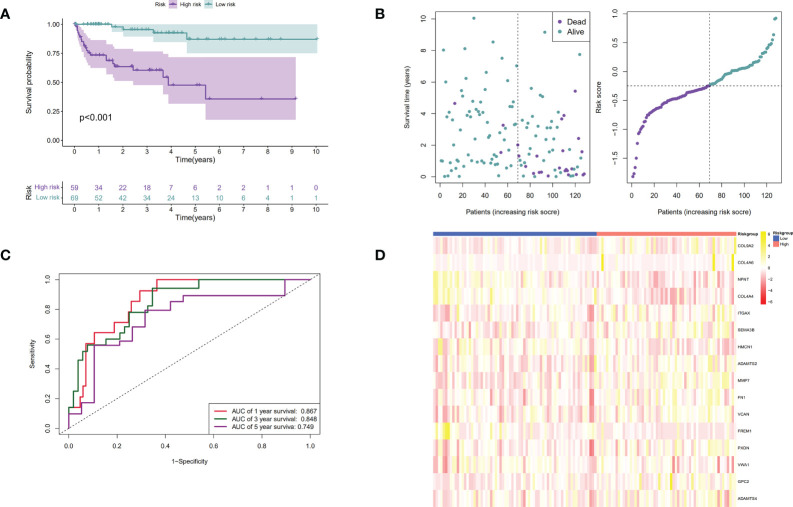
Verification of the prognostic signature by utilizing the GEO dataset. **(A).** The Kaplan-Meier curves of low-risk and high-risk ccRCC patients in the GEO dataset; **(B).** The time-dependent ROC curves displaying the 1-year, 3-year, and 5-year OS of ccRCC patients in the GEO dataset; **(C).** Survival distributions of the GEO dataset determined according to the median risk score; **(D).** Heatmap displaying the divergences between low and high-risk patients of 16 signature genes in the prognostic model for the GEO dataset.

Patients were then assigned to high-risk and low-risk groups based on the median risk score. As revealed by the Kaplan–Meier analysis, high-risk patients exhibited a significantly lower survival rate compared with the low-risk ones (*P <* 0.001), suggesting a relationship between high risk score and dismal survival (**Figures 4A, C** **)**. Additionally, the area under the ROC curve (AUC) values of the signature were 0.747, 0.719, and 0.715 at 1, 3, and 5 years, respectively, indicating that our model was stability for predicting the prognosis (**Figures 4B, D** **)**. We used data from the GEO database for external validation (**Figures 5**) and observed that the risk score was inversely correlated with survival. The AUCs of time-dependent ROC were 0.867, 0.848, and 0.749 at 1, 3, and 5 years, respectively.

### BM gene-based signature could predict ccRCC prognosis independently

**Figure 6:**
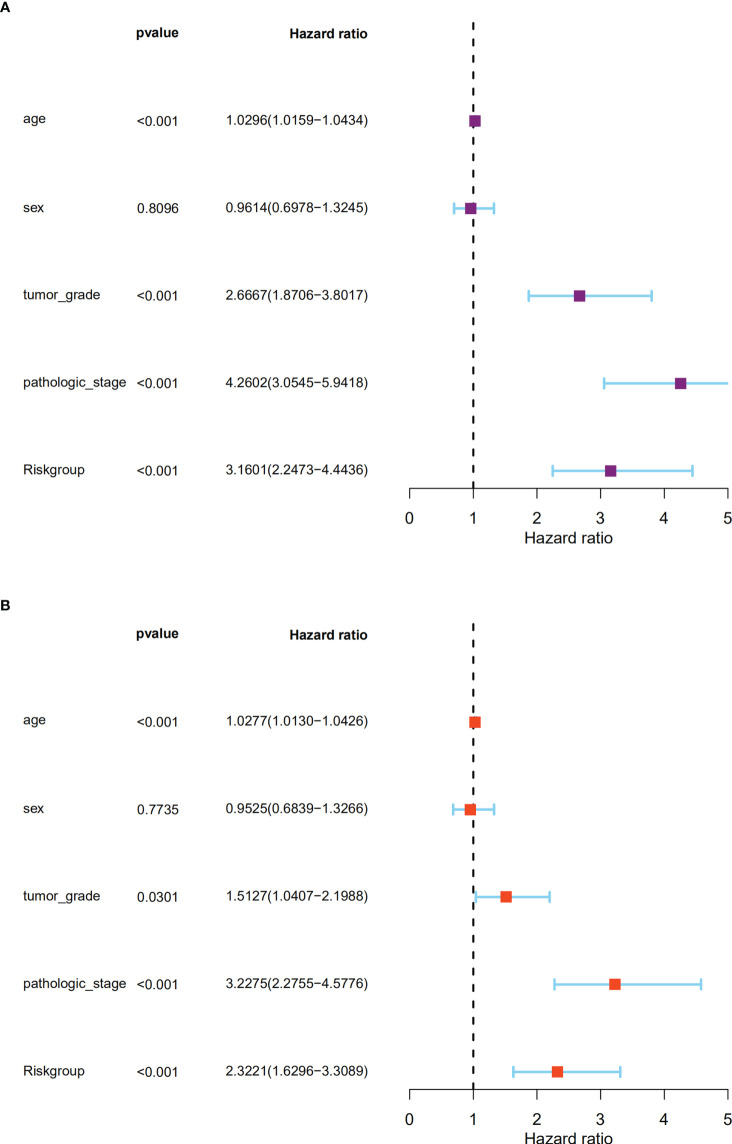
The signature could predict the prognosis of ccRCC patients in the TCGA dataset independently. **(A).** The univariate Cox regression analysis; **(B).** The multivariate Cox regression analyses showed the associations of the risk score predicting overall survival with clinicopathological indices.

The independent prognostic significance of the BM gene-based model was assessed in ccRCC patients using univariable and multivariable Cox analyses. As displayed in **Figure 6A**, univariate analysis revealed a significant correlation between age, tumor grade, pathological stage, risk score, and ccRCC patients’ survival (*P <* 0.001). Notably, the multivariate analysis also showed this correlation (*P <* 0.05) (**Figure 6B**). Therefore, based on these findings, we confirmed that our BM gene-based signature represents an independent indicator for assessing ccRCC patient prognosis.

### Relationship between clinical features and the signature

**Figure 7:**
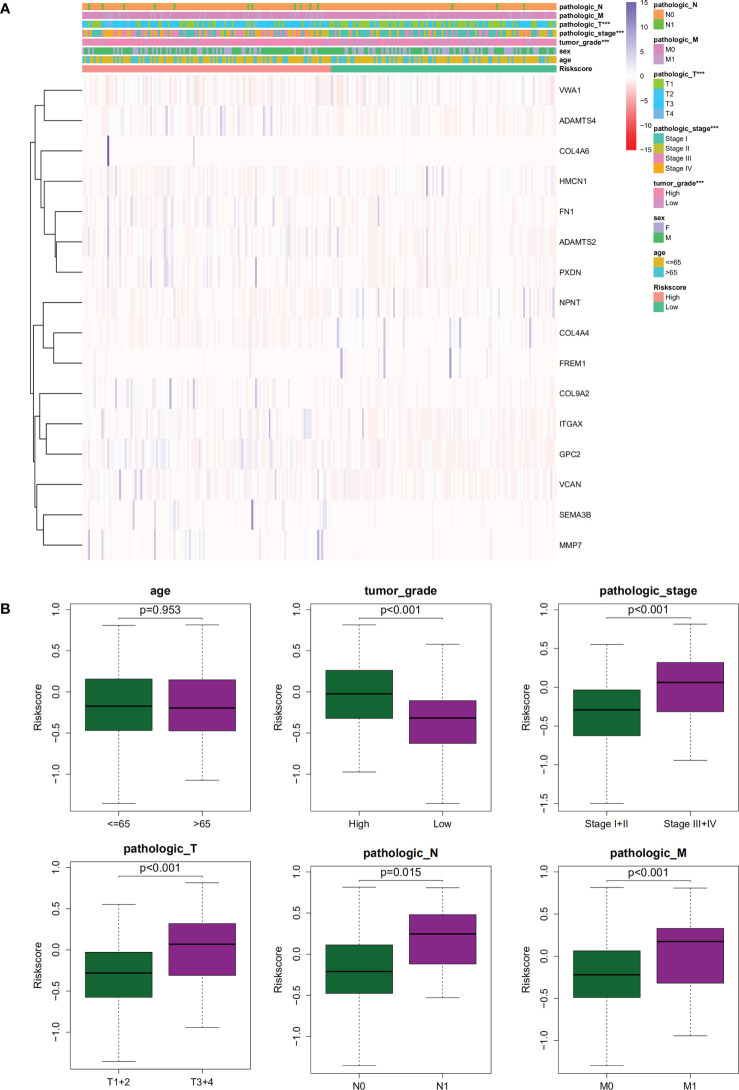
**(A, B)**. The correlations between clinicopathological features and the gene signature.

**Figure 8:**
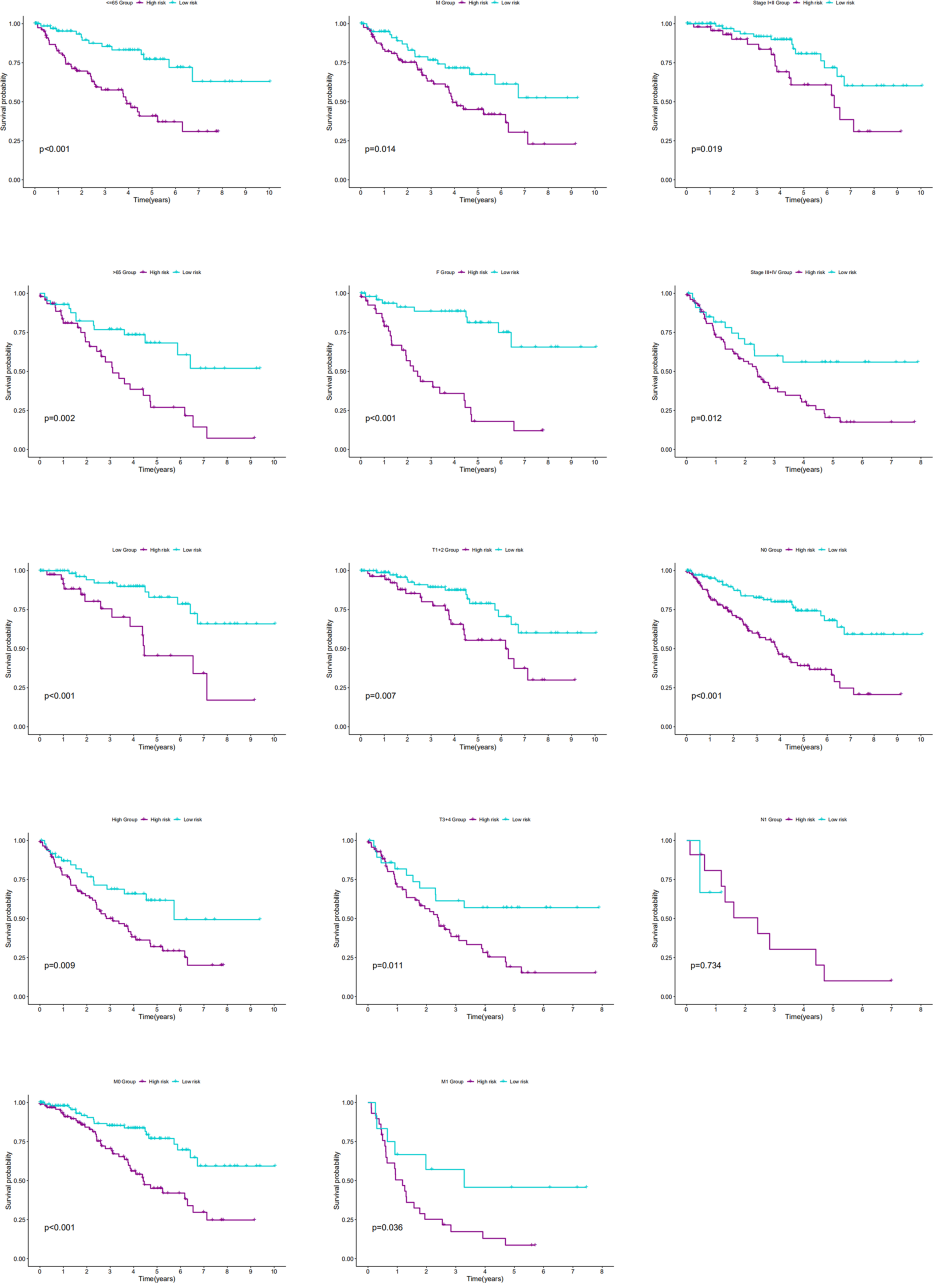
The K-M curves showed the differences of OS between low- and high-risk patients with different ages, genders, stages, T stages, N stages, M stages or grades.

The association of our signature with the progression of ccRCC was investigated using the Chi-square test. As revealed by the test, there were significant differences in the pathological stage, T stage, and tumor grade between the two groups of ccRCC patients (*P <* 0.001) (**Figures 7A,B**). Further hierarchical analysis showed the outstanding role of the model in predicting prognosis in both male and female patients (*P =* 0.0014 and *P <* 0.001, respectively), patients aged both more than, less than or equal to 65 years (*P =* 0.002 and 0.001, respectively), as well as in patients with all stages (*P =* 0.019 and 0.012 for Stages I-II and III-IV, respectively), all grades (P=0.009 and *P <* 0.001 for high and lo grades, respectively), all T stages (*P =* 0.007 and 0.011 for T1–T2 and T3–T4 stages, respectively), N0 stage (*P <* 0.001), and all M stages (*P <* 0.001 and *P =* 0.036 for M0 and M1 stages, respectively). However, the model performed poorly in predicting the prognosis for the N1 stage (P > 0.05). In the TCGA-KIRC cohort, only 15 samples were recorded with N1 stage, which might be not large enough to generate statistical significance, but the overall trend is clear that the prognostic signature deeply participated in the development and progression of ccRCC (**Figure 8**).

### Nomogram construction

**Figure 9:**
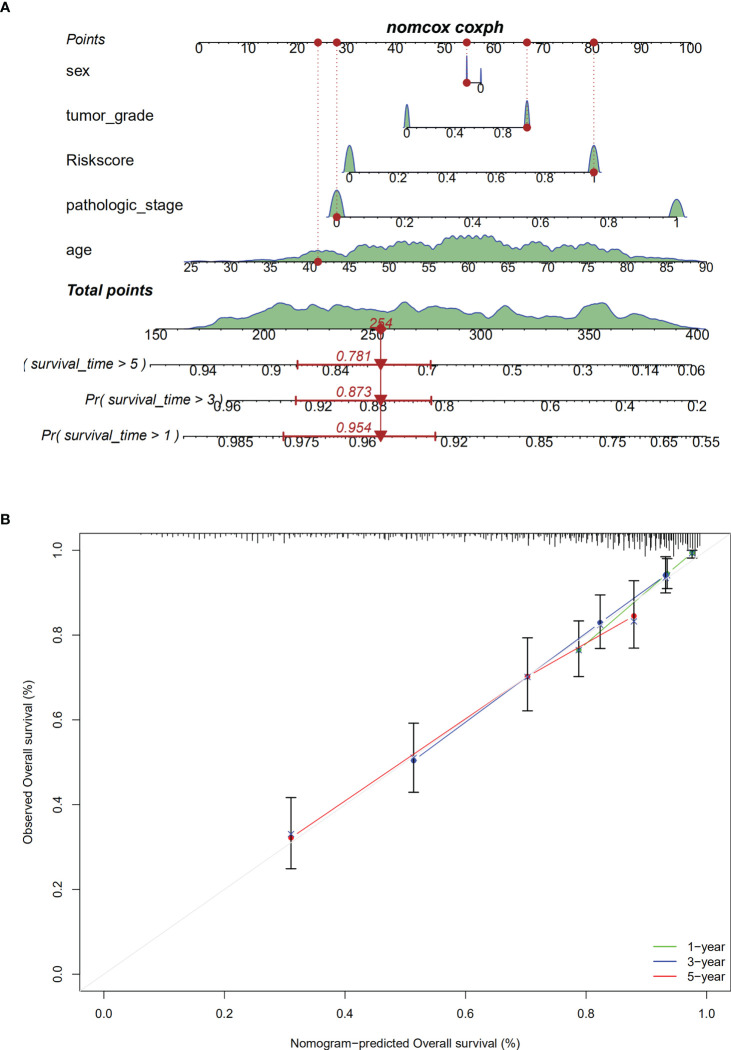
Establishment of the nomogram. **(A)**. The nomogram; **(B)**. calibration analaysis for predicting1-, 3- or 5-year OS.

We constructed a nomogram with covariates of patients’ sex, age, tumor grade, pathological stage, and risk score to predict patients’ survival rates at 1, 3, and 5 years. As shown in **Figure 9A**, each parameter has a score, and the total score was computed for survival rate prediction at the specific time point. The nomogram’s performance in survival prediction was appraised by ROC analysis. We found that the AUCs of the TCGA cohort were 0.954 for 1-year survival, 0.873 for 3-year survival, and 0.781 for 5-year survival. The calibration curve revealed the consistency of the actual survival rate of the patient with the predicted value (**Figure 9B**).

### Functional enrichment and GSEA

**Figure 10:**
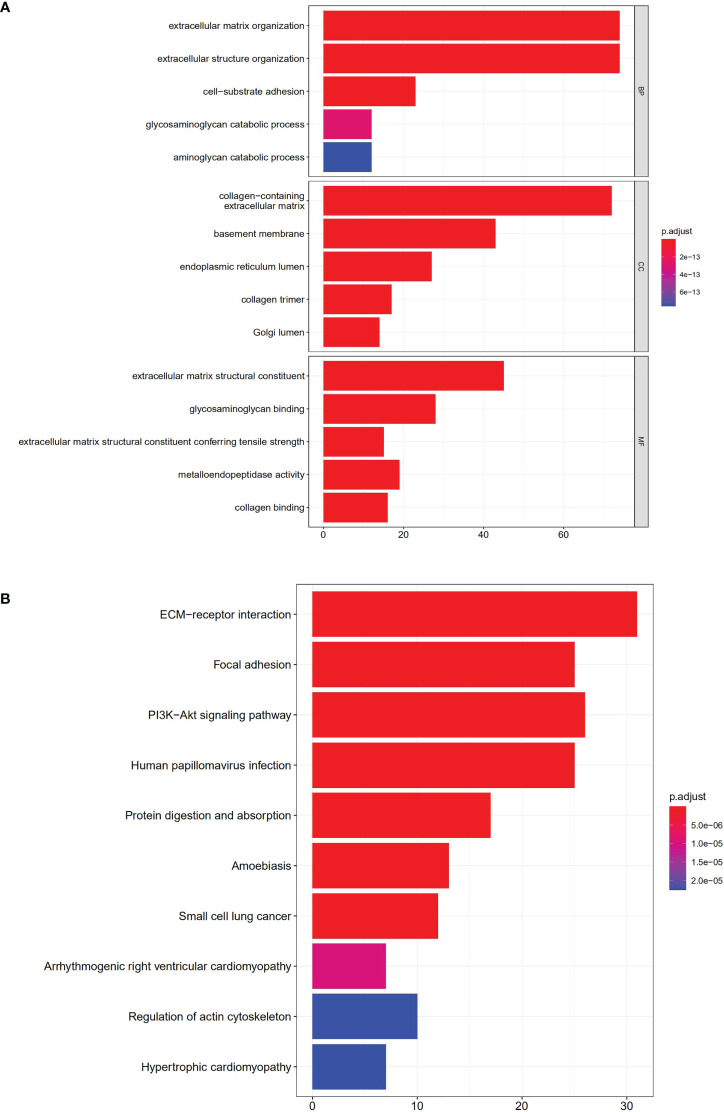
Enrichment analyses of DEGs. **(A).** GO enrichment analysis; **(B).** KEGG enrichment analysis.

GO annotation and KEGG analysis were performed to explore the potential functions of the 108 DEGs. As indicated by biological process analyses, 108 BM genes were significantly associated with the GO terms of cell–substrate adhesion, extracellular structure organization, and extracellular matrix organization. Cellular component analysis suggested that the GO terms of endoplasmic reticulum lumen, basement membrane, and collagen-containing extracellular matrix were mainly enriched. Molecular function analysis revealed that glycosaminoglycan binding, extracellular matrix structural constituent, and metalloendopeptidase activity were mainly involved in 108 DEGs (**Figure 10A**). In KEGG pathway analysis, the DEGs were primarily involved in pathways of protein digestion and absorption, PI3K/Akt signaling, focal adhesion, ECM−receptor interaction, and human papillomavirus infection (**Figure 10B**).

**Figure 11:**
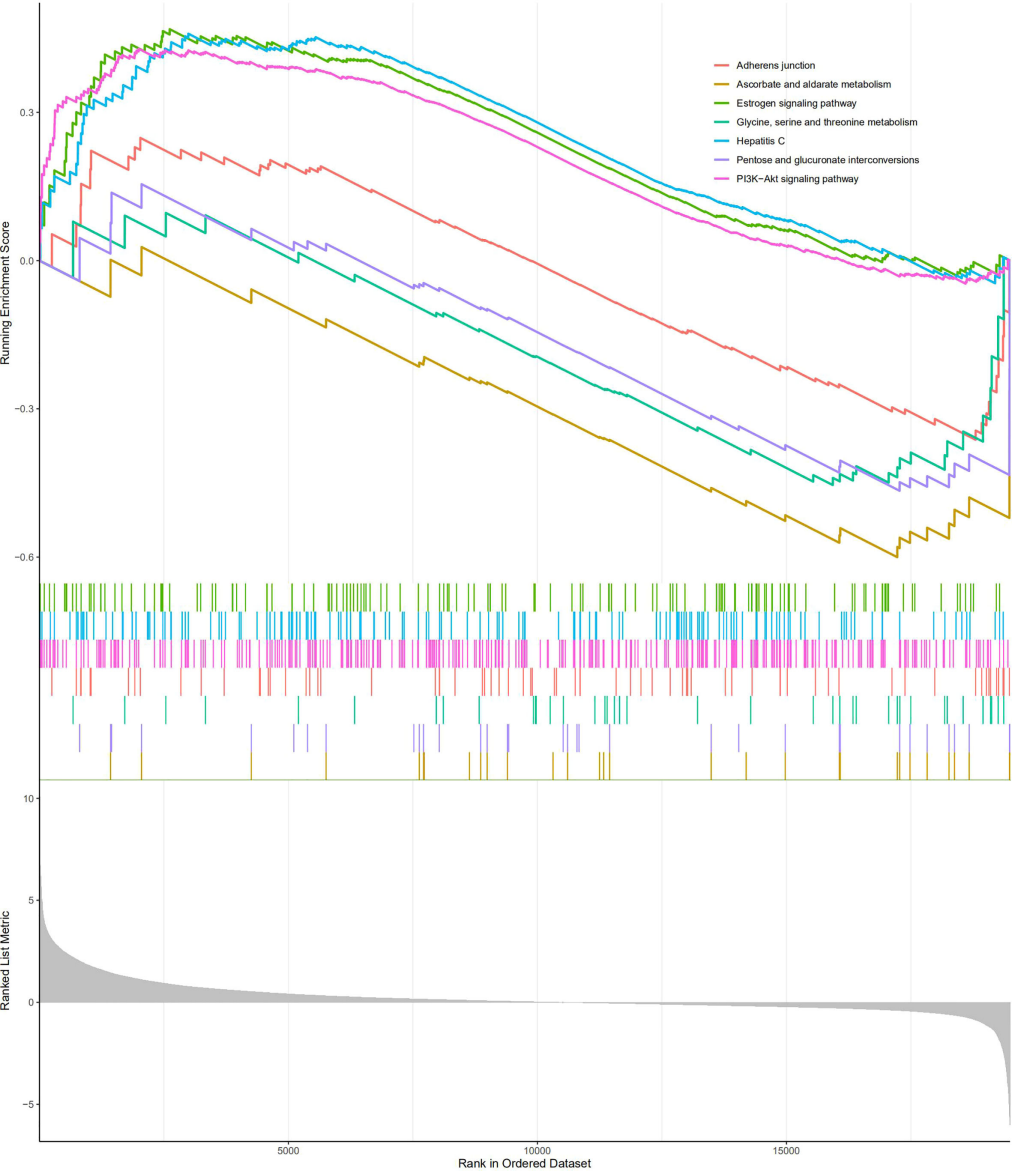
Gene Set Enrichment Analysis analysis.

GSEA was carried out to investigate the specific molecular functions of the BM gene-based model. The PI3K/Akt signaling pathway, hepatitis C pathway, and estrogen signaling pathway exhibited significant enrichment for the high-risk group; whereas for the low-risk group, the adherens junction pathway, pentose and glucuronate interconversion pathway, glycine, serine, and threonine metabolism pathways, and ascorbate and aldarate metabolism pathways were enriched (**Figure 11**).

### Analysis of the infiltration levels of immune cells based on the BM gene-based model

**Figure 12:**
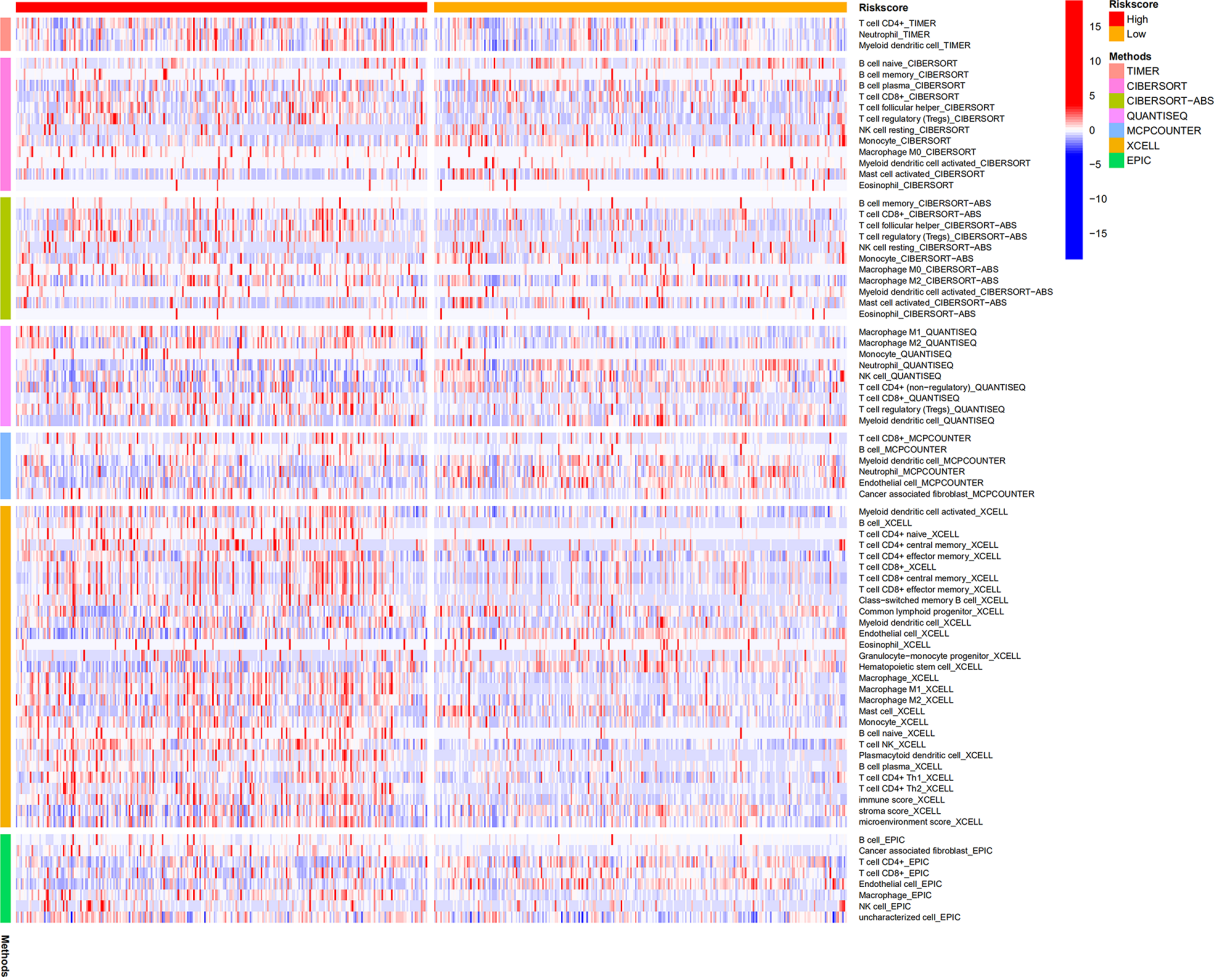
Differences in infiltration levels of immune cells between low- and high-risk patients.

**Figure 13:**
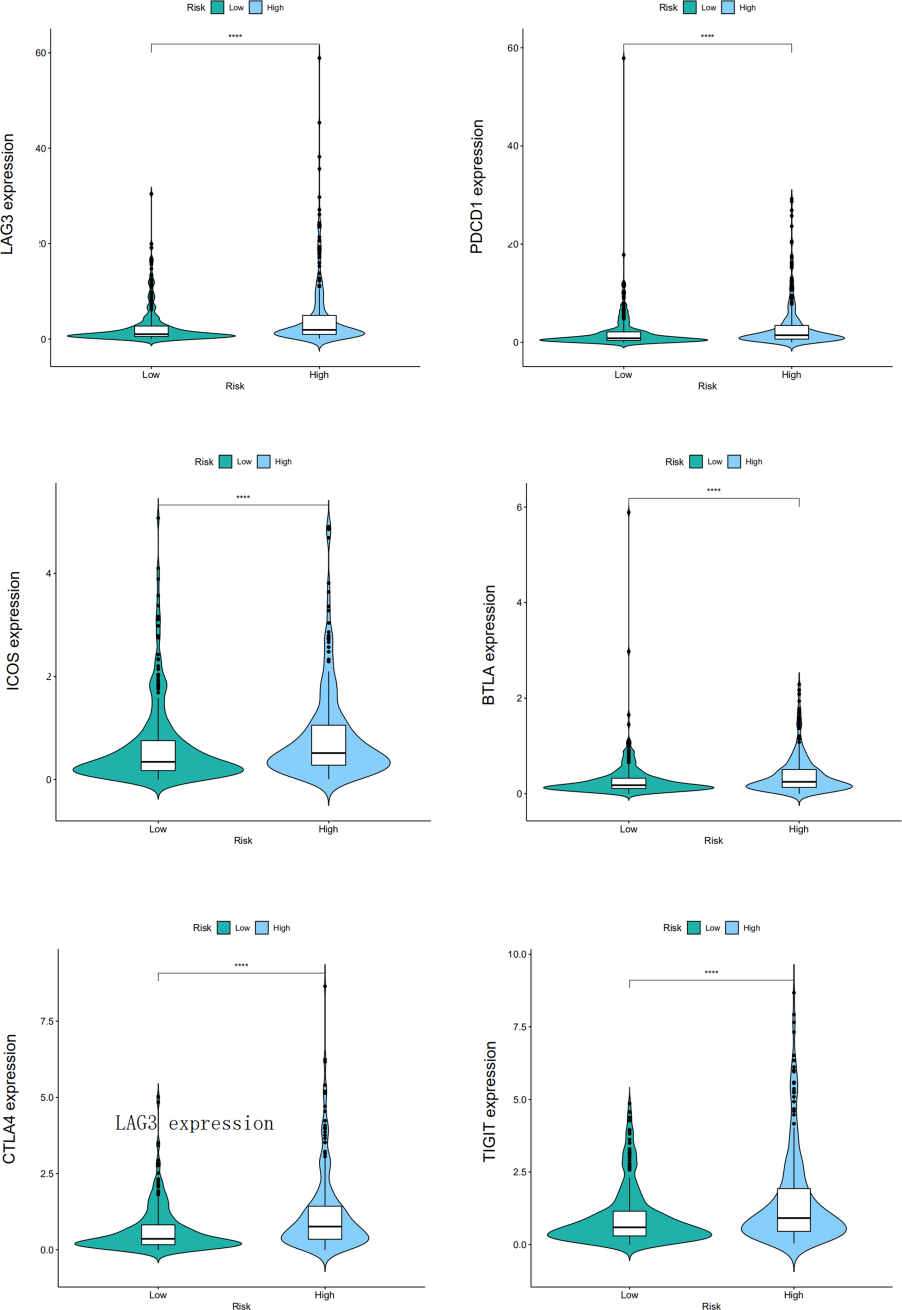
The different mRNA levels of immune checkpoint genes between low- and high-risk patients, and the "****" represents P < 0.0001.

CIBERSORT, CIBERSORT-ABS, EPIC, MCPCOUNTER, QUANTISEQ, TIMER, and XCELL analyses were performed to explore the relationship between BM gene-based signatures and immune infiltration levels (**Figure 12**). Given the significance of immune checkpoints in immunotherapy, the mRNA levels of several immune checkpoint genes were compared between the two groups to explore possible immune checkpoint blocking therapies. The results showed that *LAG3*, *PDCD1*, *ICOS*, *TIGIT*, *CTLA4*, and *BTLA* mRNA levels were increased in high-risk patients, implying the existence of immunosuppressive phenotypes in these patients (**Figure 13**).

### TIMER analysis

We explored the association of six immune cells with the 16 BM genes by employing the TIMER database and observed that *NPNT*, *COL4A6*, *ITGAX*, *HMCN1*, *ADAMTS2*, *FN1*, *VCAN*, and *PXDN* were positively associated with the levels of different immune cell infiltrations, such as those of CD4+ T cells, CD8+ T cells, B cells, dendritic cells, neutrophils, and macrophages. *COL9A2* and *ADAMTS4* were positively related to CD8+ T cells, CD4+ T cells, dendritic cells, neutrophils, and macrophages. *COL4A6* and *GPC2* exhibited positive correlations with CD4+ T cells, macrophages, neutrophils, and dendritic cells. In conclusion, these immune cells may be involved in the process of BM genes mediating ccRCC prognosis (**Supplementary Figure 1**; **Figure 2**).

### Prediction of candidate drugs implicated with the differential expression of the signature genes

We identified candidate drugs related to the differential expression of BM genes using the DSigDB to further improve the therapeutic effect in patients with renal cell carcinoma. These drugs included Healon BOSS, CGS-27023A TTD 00002801, VANADIUM CTD 00006979, LAMININ BOSS, O-Phospho-L-tyrosine BOSS, Tetradioxin CTD 00006848, endosulfan CTD 00005896, and orphenadrine hydrochloride BOSS (**Table 2**). These small-molecule drugs exhibited a higher negative correlation and potential to treat ccRCC.

**Table 2:** The eight candidate small molecule drugs predicted based on DSigDB.

Index	Name	p-value	Adjusted p-value	Odds Ratio	Combined Score
1	Healon BOSS	0.00000179	0.0007	59.68	789.76
2	CGS-27023A TTD 00002801	0.0000466	0.0095	259.39	2587.35
3	VANADIUM CTD 00006979	0.000211	0.0282	31.36	265.34
4	LAMININ BOSS	0.00048	0.0328	23.54	179.91
5	O-Phospho-L-tyrosine BOSS	0.000807	0.0438	55.83	397.64
6	Tetradioxin CTD 00006848	0.000936	0.0438	5.550	38.70
7	endosulfan CTD 00005896	0.001033676	0.0438	49.07	337.40
8	Orphenadrine hydrochloride BOSS	0.001175147	0.0438	45.90	309.68

## Discussion

Treating advanced RCC with drugs has always been a clinical challenge based on its resistance to traditional radiotherapy and chemotherapy (30). Despite the initial positive effects of emerging targeted therapies and immunotherapy in ccRCC patients, in most cases, patients develop drug resistance and disease progression within two years owing to the highly dynamic, adaptive, and heterogeneous tumor microenvironment of ccRCC (31). Therefore, research on tumor resistance and distant metastasis caused by changes in the tumor microenvironment environment may provide new strategies for ccRCC treatment. Previous research acknowledges BM remodeling as a critical step in the formation of the tumor microenvironment (32), which often results in complex disarray of pro- and anti-tumor signals from degradation products (33). Additionally, studies have demonstrated that most BM-related collagens are upregulated at the mRNA and protein levels, are associated with the formation of aggressive phenotypes of malignant cells and are involved in the regulation of key tumorigenesis steps, including proliferation, invasion, metastasis, apoptosis, and angiogenesis (34–36). Therefore, BM may genes exert crucial effects on the formation of a highly heterogeneous tumor microenvironment in ccRCC and can serve as disease markers for prognosis and treatment effect prediction in patients with renal cancer.

A prognostic model was constructed that contains 16 BM genes, and its prognostic value for ccRCC was evaluated *via* ROC anlysis. Some of these genes are potentially related to ccRCC. For instance, MMP7 has been widely reported to promote tumor angiogenesis by transforming the extracellular matrix, thereby participating in the invasion and metastasis of ccRCC (37–39). A previous study identified SEMA3B as a renal tumor suppressor gene, whose downregulation was positively associated with tumor progression, stage, and grade of ccRCC (40). As a vital member of the BM gene family, *ITGAX* is responsible for encoding integrin alpha X, a critical component of leukocyte-specific complement receptor 4. Its expression in ccRCC has been reported to increase significantly, and ITGAX overexpression has association with dismal survival outcomes of ccRCC patients (41). Gong et al. recently reported that the *HMCN1* mutations are frequently detected in patients with ccRCC and are correlated with a higher tumor mutation burden and dismal clinical consequences, and may correlate with anti-tumor immunity and cell metabolism (42). In addition, *COL4A4* has been identified as an unfavorable prognostic factor for ccRCC (43). The functions of other genes in ccRCC currently remain unknown and require further exploration. Data from the TGGA and GSEA databases indicated that the BM gene signatures were positively correlated with a higher risk of adverse OS. Meanwhile, the AUCs were all above 0.7 at 1, 3, and 5 years. These results indicated the admirable performance of our model for prognosis prediction.

According to KEGG pathway enrichment analysis, focal adhesions and ECM-receptor interactions were identified as the major pathways for 108 DEGs. These pathways further enriched the molecular mechanisms of ccRCC initiation and progression. GSEA revealed the involvement of BM gene-based models in tumor and metabolic pathways. These include the PI3K/Akt signaling, estrogen signaling, adherens junction, pentose and glucuronate interconversions, threonine, glycine and serine metabolism, and ascorbate and aldarate metabolism pathways. Therefore, the BM gene-based model may be crucial for cancer cell metabolism and tumor microenvironment formation.

Furthermore, the model had close association with immune cell infiltration, immune cells may be essential in BM genes mediating the prognosis of ccRCC. We also found higher expression levels of immune checkpoints in high-risk ccRCC patients, implying that the dismal prognosis of these patients is possibly due to the immunosuppressive microenvironment and may respond to treatment regimens involving checkpoint inhibitors. Finally, given that the signature BM genes we identified may be relevant therapeutic targets for patients with ccRCC, we sucessfully dentified eight potential small-molecule drugs to further improve the therapeutic effect in patients with ccRCC.

Our work has certain limitations, such as predicting the prognostic value of BM genes using only data from public databases and the relatively small sample size. We could only determine how BM genes affect ccRCC based on limited clinical data, which ignored environmental and genetic factors. Finally, the underlying mechanism between the identified signature genes and ccRCC remains unclear, and we plan to investigate this further.

In summary, this study comprehensively characterized the involvement of the BM gene family in ccRCC and its prognosis. We proposed trustworthy prognostic biomarkers for ccRCC patients and constructed a BM gene-based prognostic model. We believe this investigation could support further research on the role of BM genes in ccRCC.

## Data availability statement

The original contributions presented in the study are included in the article/**Supplementary Material**. Further inquiries can be directed to the corresponding authors.

## Ethics statement

The studies involving human participants were reviewed and approved by The Ethics Review Board of the First Affiliated Hospital of Anhui Medical University. Written informed consent for participation was not required for this study in accordance with the national legislation and the institutional requirements.

## Author contributions

JT and XL conceived the experiments and performed the experiments and drafted the manuscript, CL provided supportive advice for the experiment, YL and JZ confirmed the authenticity of all the raw data and funded the research. All authors contributed to the article and approved the submitted version.

## Funding

This work was supported by Natural Science Foundation of Anhui Province in China (No. 2108085MH295).

## Conflict of interest

The authors declare that the research was conducted in the absence of any commercial or financial relationships that could be construed as a potential conflict of interest.

## Publisher’s note

All claims expressed in this article are solely those of the authors and do not necessarily represent those of their affiliated organizations, or those of the publisher, the editors and the reviewers. Any product that may be evaluated in this article, or claim that may be made by its manufacturer, is not guaranteed or endorsed by the publisher.
