# Supplementary figures and images for: Expression of basement membrane genes and their prognostic significance in clear cell renal cell carcinoma patients
Source: Front Oncol. 2022 Oct 24;12:1026331. doi: 10.3389/fonc.2022.1026331 (PMC9637577; doi:10.3389/fonc.2022.1026331)

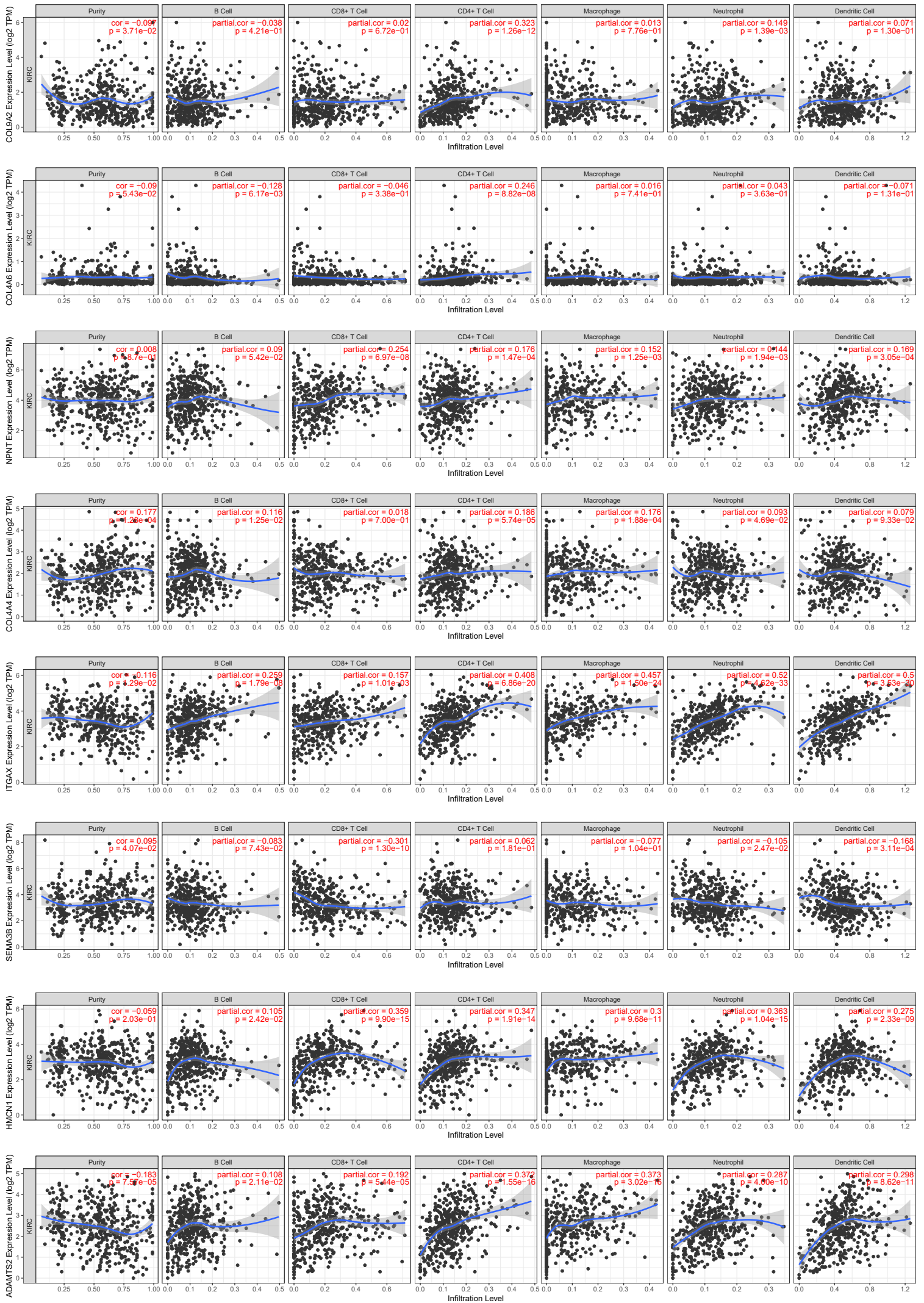

Supplement: Supplementary file 1 [file Image_1.pdf]

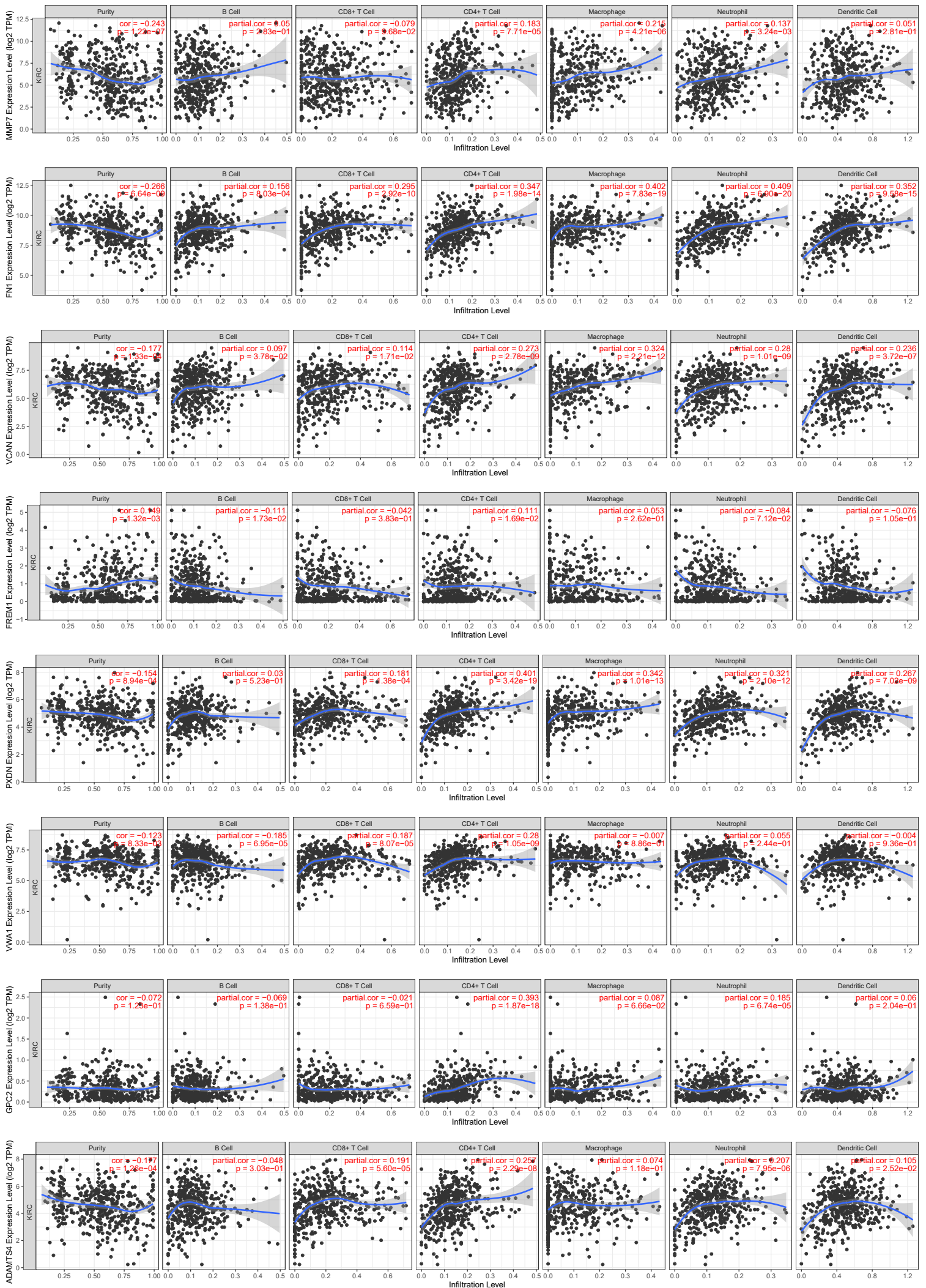

Supplement: Supplementary file 2 [file Image_2.pdf]
