# Supplementary material for: Expression of basement membrane genes and their prognostic significance in clear cell renal cell carcinoma patients
Source: Front Oncol. 2022 Oct 24;12:1026331. doi: 10.3389/fonc.2022.1026331 (PMC9637577; doi:10.3389/fonc.2022.1026331)
Supplement: Supplementary file 3 [file Table_1.pdf]

Supplementary Table 1 Primers used in the present study

Gene	Primers sequences
GAPDH	F: ACAACTTTGGTATCGTGGAAGG
	R: GCCATCACGCCACAGTTTC
COL9A2	F: CTCGCTCTGGCGCAGATTAG
	R: GCCCATTGTCACCGTCGAT
COL4A6	F: CAGCAGCGGGAGAGAAGTC
	R: CCAGTAGAGCCAGTGAATCCT
COL4A4	F: AGGCTCAACTGGTCTAAGAGG
	R: GCAGGGTCACCTTTGTTTCC
NPNT	F: GTAAGCACAGGTGCATGAACA
	R: GAACCATCCGGCATGAGCATA
ITGAX	F: AGAGCTGTGATAAGCCAGTTCC
	R: AATTCCTCGAAAGTGAAGTGTGT
SEMA3B	F: GGGAGGAGCTATACTCAGGGG
	R: TTCGGAGACTTGGACGTTGC
ADAMTS2	F: GACACGGGCCACGATGAATA
	R: GGTGACAGGAGCATAGCCTT
FREM1	F: GCCTGTGGTAACCAGGAACAA
	R: CGCAGGTGTATCAGGGTCG
ADAMTS4	F: GAGGAGGAGATCGTGTTTCCA
	R: CCAGCTCTAGTAGCAGCGTC
PXDN	F: AATCAGAGAGATCCAACCTGGG
	R: AATGCTCCACTAGGTATCCTCTT
